# Notes from Scandinavia

**Published:** 1889-12-28

**Authors:** 


### Notes from Scandinavia.

(*By Our Copenhagen Corrspondent.*)

At Hunnerup, close to the ancient town of Lund, in West Sweden, a very large asylum is approaching its completion. It has been built quite close to what has been hitherto known as the Kjelby Hospital, and the two together will be called the Lund Hospital. The asylum for incurable lunatics comprises some seven or eight buildings; they are built of red brick, and the three largest buildings are four storeys high. In these buildings there is accommodation for 685 patients, besides apothecary, and church, and residences for doctors and attendants. The grounds are between thirty and forty acres at Hunnerup and about twice this area at Kjelby, the two being divided by a small river, over which an iron bridge has been built. A large proportion of the grounds will be transferred into gardens and shrubberies. The whole concern will be able to accommodate 1,035 patients, and Dr. Svante Odenan, of Lund, will be the chief physician.

Prof. Kraft Ebing has been appointed first professor at the asylum at Vienna, and delivered the other day a lecture on his entering into his new function. He pointed out the important advances the treatment of lunatics had made in the present century. In civilised countries there is now about one lunatic to every two hundred persons (adult). Of the lunatics, about 56 per cent. in the poorer classes, but only about 16 per cent. in the better classes were cured, the difference arising out of the fact that poor people generally were sent to the asylums at once, whereas more well-to-do people generally tried a number of ineffective means before going there. Prof. Ebing finally pointed out that hypnotism was possibly destined to direct the study of lunacy into entirely new paths.

The Danish capital, which already boasts some exceedingly fine and well-arranged infirmaries, is likely to soon see another large institution added to their number, the Church Minister having laid a proposal to this effect before the Danish Parliament. It is proposed to discontinue the use of the "Frederick's Infirmary" with the adjoining institution for the confinement of women of the poorer classes, and to erect a large new infirmary on one of the commons outside the town, but facing a large thoroughfare, and at a convenient distance for some of the largest and most populous parts of the town. The position is very open and well suited for the purpose, and the grounds will comprise some forty to fifty acres, which is about seven times larger than the area of the two institutions, which will be removed to the new infirmary. It proposed to adopt the "pavilion system," with a few large and a number of smaller detached buildings, in some cases connected by covered corridors, and surrounded with gardens and shrubberies. The infirmary proper will have 816 beds as compared with 381 at the present Frederick's Infirmary. Connected with the infirmary will be the following specialist consulting establishments: one for medicine, one for surgery, one for female complaints, one for complaints of the eye, one for nose, throat, and ear complaints, one for syphilis and skin diseases, one psychiatric section and one for children's diseases. The institution for confinements will contain 106 beds, the nursing establishment accommodation for 51 children, with 43 wet nurses. There will also be a school for sick nurses, where ladies of the better classes can acquire the necessary knowledge, and finally there will be erected three separate buildings for the use of the medical college. The three buildings will be used as a physiological, an anatomical, and a pathological institution.

It is proposed to extend the working of the institution for confinements by offering accommodation for thirty-two women of the poorer classes in the last months of their pregnancy, on the conditions that they do a certain amount of work and may be used at the instruction of the midwife pupils and the students. This arrangement is expected to answer very well, giving good material for important studies, and offering relief to a number of poor women, who might otherwise become so reduced that they might have some trouble in getting over their confinement.

The building of the infirmary should be completed in five years, and should not be commenced later than during the year 1892. The total cost of buildings, etc., has been calculated at 5,800,000kr., or about £325,000, but the site of the old infirmary is valued at more than £150,000, and the infirmary should of its own capital contribute about £50,000, so the State would only have to contribute rather more than £100,000. A naval infirmary and a large institution for public gymnastics are also under contemplation.

